# Phase 1 and pharmacokinetic study of LY3007113, a p38 MAPK inhibitor, in patients with advanced cancer

**DOI:** 10.1007/s10637-017-0532-2

**Published:** 2017-12-01

**Authors:** Jonathan W. Goldman, Lee S. Rosen, Anthony W. Tolcher, Kyriakos Papadopoulos, Muralidhar Beeram, Peipei Shi, Celine Pitou, Robert Bell, Palaniappan Kulanthaivel, Xuekui Zhang, Aaron Fink, Edward M. Chan, Ashwin Shahir, Daphne Farrington, Amita Patnaik

**Affiliations:** 1 University of California, Los Angeles, Santa Monica, CA USA; 2 South Texas Accelerated Research Therapeutics, San Antonio, TX USA; 3 START Center for Cancer Care, San Antonio, TX USA; 4 Eli Lilly & Company, Indianapolis, IN USA; 5 Present Address: Biogen Inc., Washington, DC USA; 6 Present Address: AstraZeneca Pharmaceuticals LP, Gaithersburg, MD USA; 7 Present Address: Genentech, San Francisco, CA USA

**Keywords:** p38 mitogen-activated protein kinase, Advanced cancer, Inhibitor

## Abstract

*Background* The signaling protein p38 mitogen-activated protein kinase (MAPK) regulates the tumor cell microenvironment, modulating cell survival, migration, and invasion. This phase 1 study evaluated the safety of p38 MAPK inhibitor LY3007113 in patients with advanced cancer to establish a recommended phase 2 dose. *Methods* In part A (dose escalation), LY3007113 was administered orally every 12 h (Q12H) at doses ranging from 20 mg to 200 mg daily on a 28-day cycle until the maximum tolerated dose (MTD) was reached. In part B (dose confirmation), patients received MTD. Safety, pharmacokinetics, pharmacodynamics, and tumor response data were evaluated. *Results* MTD was 30 mg Q12H. The most frequent treatment-related adverse events (>10%) were tremor, rash, stomatitis, increased blood creatine phosphokinase, and fatigue. Grade ≥ 3 treatment-related adverse events included upper gastrointestinal haemorrhage and increased hepatic enzyme, both occurring at 40 mg Q12H and considered dose-limiting toxicities. LY3007113 exhibited an approximately dose-proportional increase in exposure and time-independent pharmacokinetics after repeated dosing. Maximal inhibition (80%) of primary biomarker MAPK-activated protein kinase 2 in peripheral blood mononuclear cells was not reached, and sustained minimal inhibition (60%) was not maintained for 6 h after dosing to achieve a biologically effective dose (BED). The best overall response in part B was stable disease in 3 of 27 patients. *Conclusions* The recommended phase 2 dosage of LY3007113 was 30 mg Q12H. Three patients continued treatment after the first radiographic assessment, and the BED was not achieved. Further clinical development of this compound is not planned as toxicity precluded achieving a biologically effective dose.

## Introduction

The signaling protein p38 mitogen-activated protein kinase (MAPK) is activated by cancer cells downstream of oncogenic receptor tyrosine kinases and in response to both radiation and chemotherapy [1]. It phosphorylates a number of substrates, including MAPK-activated protein kinase 2 (MAPKAP-K2), and regulates the production of key cytokines in the microenvironment, such as tumor necrosis factor α, interleukin-1β,interleukin-6 (IL-6), and interleukin-8 [2–4]. These cytokines are up-regulated in many human malignancies and promote cancer cell survival, growth, invasion, and metastasis [4, 5], including non–small cell lung cancer, ovarian cancer, glioblastoma, multiple myeloma, lymphoma, breast cancer, and colon cancer.

Recent studies have indicated that p38 MAPK down-regulates fibulin 3 expression and leads to migration and invasion in some cancers [6]. Furthermore, p38 MAPK plays a role in angiogenesis and may be inhibited to reduce the secretion of cytokines that promote angiogenesis [7]. As it relates to the tumor microenvironment, p38 MAPK controls the senescence-associated secretory phenotype and, when inhibited, mitigates against the tumor-forming activities of the cancer associated fibroblasts [8]. Thus, pharmacologic inhibition of p38 MAPK directed to both the cancer and its supportive microenvironment represents a novel therapeutic strategy for improving outcomes for individuals with these cancers.

In p38 MAPK knockout mice, phosphorylated MAPK-activated protein kinase 2 (p-MAPKAP-K2) levels are significantly reduced, and circulating tumor necrosis factor α and IL-6 levels are greatly attenuated [9]. This phenotype is remarkably similar to that observed in MAPKAP-K2–deficient mice [3]. Interestingly, inhibition of p38 MAPK not only reduces secretion of IL-6 and vascular endothelial growth factor in bone marrow stromal cells, but also inhibits growth of human multiple myeloma cells [10]. In preclinical studies, LY3007113 inhibited phosphorylation of MAPKAP-K2 in HeLa cells, indicating that this small-molecule inhibitor of p38 MAPK has intracellular activity. In mice, orally administered LY3007113 has been observed to inhibit p-MAPKAP-K2 in peripheral blood and in human gliablastoma tumors (U87MG) implanted subcutaneously. In addition, LY3007113 has shown activity when administered alone in xenograft models of human ovarian and kidney cancers and leukemia.

The primary objective of this study was to evaluate the safety and tolerability of LY3007113 when administered orally to patients with advanced cancer to establish a recommended dose for phase 2 studies. The secondary objectives were to assess the pharmacokinetics (PK), pharmacodynamics (PD), and tumor response rate in patients who received LY3007113.

## Methods

### Study design and patients

This multicenter, nonrandomized, open-label, dose-escalation phase 1 study had 2 parts: dose escalation (part A) and dose confirmation (part B).The study enrolled adults aged 18 years or older with histologic or cytologic evidence of advanced or metastatic cancer. Other inclusion criteria included an Eastern Cooperative Oncology Group performance status score of ≤2 and adequate organ function. Exclusion criteria included major surgical resection involving the stomach or small bowel, symptomatic central nervous system malignancy or metastasis, diagnosis of acute leukemia, history of any other cancer (except nonmelanoma skin cancer or carcinoma in situ of the cervix) unless in complete remission and not treated for at least 3 years, or an autologous or allogeneic stem-cell transplant within 75 days of starting study drug. The study was conducted in accordance with the Declaration of Helsinki and good clinical practice guidelines, and the protocol was approved by each participating institution’s ethics review board. All patients provided written informed consent.

In part A, LY3007113 was to be administered orally at dosages ranging from 20 mg to 200 mg every 12 h (Q12H) daily on a 28-day cycle, with modifications during cycle 1 (ie, drug dosing on day −3) to enable PK sampling for 72 h after a single dose. Dose escalation was guided by safety assessments during cycle 1 using the Common Terminology Criteria for Adverse Events, Version 4.0, and continued until the maximum tolerated dose (MTD) (ie, the highest dose level at which <33% of patients experienced a dose-limiting toxicity [DLT] event during cycle 1 was reached. A DLT was defined as an adverse event (AE) that was possibly related to the study drug and met any of the following criteria: grade ≥ 3 nonhematological toxicity event (except nausea, vomiting, diarrhea, or electrolyte disturbance); grade ≥ 3 nausea, vomiting, diarrhea, or electrolyte disturbance lasting more than 2 days despite maximal supportive intervention; grade 4 hematological toxicity event lasting more than 5 days; grade ≥ 3 thrombocytopenia with bleeding (except epistaxis); or grade ≥ 3 febrile neutropenia. If a DLT occurred in more than 1 patient at any dose level, dose escalation ceased at that dose level, and the previous dose was declared to be the MTD.

After the last patient in part A had completed cycle 1 and the MTD was determined, the dose-confirmation phase (part B) began. In part B, approximately 15 evaluable patients were treated with the MTD on days 1 through 28 of a 28-day cycle, with modifications during cycle 1 to enable PK sampling after a single dose and repeated doses.

Patients received 2 cycles of LY3007113 unless they met one or more of the criteria for discontinuation. Patients who experienced clinical benefit, in the investigator’s opinion, were permitted to receive additional cycles.

LY3007113 was supplied by Eli Lilly and Company (Indianapolis, IN) as 10-mg and 40-mg capsules for oral administration.

### Pharmacokinetic studies

Plasma concentrations were determined for LY3007113 and its metabolites, LSN3025641 and LSN3047151, using a validated liquid chromatography with tandem mass spectrometry method. The analytes were extracted from human plasma by protein precipitation and chromatography was performed using an Onyx Monolithic C18 column (Phenomenex). Analysis was performed with positive ion electrospray for the analytes. For LY3007113, the lower and upper limits of quantification were 1 ng/mL and 1000 ng/mL. Interassay accuracy (percent relative error) during validation ranged from −0.9% to 7.0% for LY3007113, from 0.2% to 6.0% for LSN3025641, and from −1.4% to 4.0% for LSN3047151. Interassay precision (percent relative SD) during validation ranged from 2.8% to 5.6% for LY3007113, from 3.2% to 7.5% for LSN3025641, and from 3.9% to 8.7% for LSN3047151. Plasma sample analysis was conducted at Covance Bioanalytical Services, LLC (Indianapolis, IN).

The PK analyses included but were not limited to maximum plasma concentration (C_max_), area under the concentration-versus-time curve (AUC), terminal half-life (t_1/2_), apparent volume of distribution, apparent clearance, and other relevant parameters calculated after administration of the first dose on day −3 as single-dose PK and then on days 1, 14, and 28 of cycle 1.

The PK parameter estimates of C_max_ and AUC for LY3007113 were evaluated statistically to delineate the effects of dose proportionality using methods described previously [9]. Least-squares estimates of geometric means and corresponding 90% confidence intervals (CIs) were provided by dose with the dose-normalized ratio of geometric means and CI.

Intracellular levels of p-MAPKAP-K2 were measured before and after administration of LY3007113 by using flow cytometry after ex vivo stimulation of peripheral blood mononuclear cells (PBMCs) from patients, 20 μg/ml of anisomycin for 20 min. The biologically effective dose (BED) was defined as the lowest dose that would achieve at least 80% maximal inhibition of p-MAPKAP-K2 and at least 60% inhibition for up to 6 h after dose in PBMCs.

### Tumor response

Tumors were radiographically assessed according to Response Evaluation Criteria in Solid Tumors, Version 1.1, at baseline (day −28 to day −4) and in every other cycle beginning with cycle 2 before the start of the subsequent cycle for patients in part B. The overall response rate (ie, percentage of patients with best response of either complete response or partial response) was determined for patients in Part B only.

## Results

### Patient demographics, dosing, and disposition

Twenty-seven patients (12 patients in part A and 15 patients in part B) were enrolled and received at least 1 dose of study drug. Table 1 summarizes patient demographics and disease characteristics at baseline. Patients were heavily pre-treated and exhausted all available therapies. Primary tumor types identified in 2 or more patients included colon, pancreas, rectal, adrenocortical, and breast.

**Table 1:** Patient demographics and baseline characteristics

	Part A (*N* = 12)				Part B (*N* = 15)
	Cohort 1 (20 mg Q12H) (*n* = 3)	Cohort 2 (40 mg Q12H) (*n* = 4)	Cohort 3 (30 mg Q12H) (*n* = 5)	Total (*n* = 12)	
Sex, *n* (%)					
Female	2 (66.7)	3 (75.0)	2 (40.0)	7 (58.3)	8 (53.3)
Male	1 (33.3)	1 (25.0)	3 (60.0)	5 (41.7)	7 (46.7)
Age, years					
Mean	60.3	63.8	56.2	59.8	59.4
Range	56–65	57–69	48–75	48–75	44–73
Race, *n* (%)					
Asian	1 (33.3)	0	2 (40.0)	3 (25.0)	0
Black/African American	0	0	0	0	2 (13.3)
White	2 (66.7)	4 (100.0)	3 (60.0)	9 (75.0)	13 (86.7)
ECOG PS, *n* (%)					
0	0	1 (25.0)	3 (60.0)	4 (33.3)	5 (33.3)
1	3 (100.0)	3 (75.0)	2 (40.0)	8 (66.7)	10 (66.7)
≥ 2	0	0	0	0	0
Primary tumor type					
Adenocarcinoma, colon	1 (33.3)	3 (75.0)	0	4 (33.3)	0
Adenocarcinoma, gastric	0	0	0	0	1 (6.7)
Adenocarcinoma, pancreas	1 (33.3)	0	1 (20.0)	2 (16.7)	0
Adenocarcinoma, prostate	0	0	0	0	1 (6.7)
Adenocarcinoma, rectum	1 (33.3)	0	0	1 (8.3)	2 (13.3)
Carcinoma, urothelium	0	0	1 (20.0)	1 (8.3)	0
Carcinoma, adrenocortical	0	0	1 (20.0)	1 (8.3)	1 (6.7)
Carcinoma, breast	0	0	0	0	2 (13.3)

*Abbreviations*: *ECOG*, Eastern Cooperative Oncology Group; *PS*, performance status; *Q12H*, every 12 h

In part A, patients received LY3007113 dosages of 20 mg (*n* = 3), 30 mg (*n* = 4), or 40 mg (*n* = 5) Q12H. The mean (SD) numbers of received cycles and completed cycles (ie, all scheduled doses in a cycle received) were 1.3 (0.87) and 0.5 (0.67) respectively (ranges, < 1 to 2 in both cases),. The majority of patients did not complete the first cycle.

In part B, 15 patients received an LY3007113 dose of 30 mg Q12H (60 mg daily). The mean (SD) numbers of received cycles and completed cycles were 1.4 (6.3) and 0.4 (0.63), respectively (ranges, < 1 to 2 in both cases).

Overall, 5 patients (18.5%) received more than 2 cycles of treatment: 1 patient (renal cell carcinoma) received 3 cycles, 3 patients (pancreas adenocarcinoma, adrenocortical carcinoma, and epithelial ovarian carcinoma) received 4 cycles, and 1 patient (gastric adenocarcinoma) received 5 cycles.

Overall, 25 of 27 patients (92.5%) discontinued study drug. In part A, all 12 patients discontinued study drug: 10 patients (83.3%) had progressive disease, 1 patient (8.3%) died because of progressive disease, and 1 patient (8.3%) was discontinued because of physician decision. In part B, 13 patients (86.6%) discontinued study drug: 11 patients (84.6%) had progressive disease, 1 patient (6.7%) chose to discontinue, and 1 patient (6.7%) was discontinued due to physician decision. Two patients (13.3%) in part B did not give a reason for discontinuation of study drug.

### Safety, maximum tolerated dose, and dose determination

The most frequent treatment-emergent adverse events (TEAEs) reported in ≥10% of patients were tremor (11 patients [40.7%]); rash, including the terms rash, rash maculopapular, rash papular, and dermatitis acneiform (10 patients [37.0%]); fatigue (8 patients [29.6%]); dyspnoea, including the terms dyspnoea and dyspnoea exertional (7 patients [25.9%]); constipation and diarrhea (5 patients each [18.5%]); anaemia, nausea, peripheral edema, and stomatitis (4 patients each [33.3%]); and abdominal pain, increased blood creatine phosphokinase, decreased appetite, cough, dehydration, epistaxis, upper respiratory tract infection, hypotension, vertigo, and vomiting (3 patients each [11.1%]).

Most TEAEs were reported as mild (grade 1) or moderate (grade 2). Grade 3 TEAEs (*n* = 16) were reported in a total of 9 patients. These TEAEs included gastrointestinal haemorrhage (*n* = 3), intestinal obstruction (*n* = 2), pneumonia (*n* = 2), anaemia, peptic ulcer, pericardial effusion, unilateral blindness, constipation, abdominal pain, thrombocytopenia, hypercalcaemia, and increased hepatic enzyme. Grade 4 TEAEs (*n* = 3) were reported in 2 patients. These included sepsis and hypovolaemia with hypotension.

Study-drug–related TEAEs were reported in 18 patients (66.7%), including 10 patients (83.3%) in part A and 8 patients (53.3%) in part B (Table 2). Overall, the most frequent were tremor (9 patients [33.3%]); rash, including the terms rash maculopapular and dermatitis acneiform (6 patients [22.2%]); stomatitis (4 patients [14.8%]); and increased blood creatine phosphokinase and fatigue (3 patients each [11.1%]).

**Table 2:** Most common treatment-related adverse events (≥2 patients overall) by grade in safety population

	Part A			Part B (*N* = 15)	Grades^b^
	Cohort 1 20 mg (*n* = 3)	Cohort 2 40 mg (*n* = 4)	Cohort 3 30 mg (*n* = 5)		
Toxicities^a^					
Tremor	0	3(75%)	1(20%)	5(33%)	Grades 1, 2
Rash^c^	1(33%)	2(50%)	1(20%)	6(22%)	Grades 1, 2
Stomatitis	0	1(25%)	2(40%)	4(14.8%)	Grades 1, 2
Increased blood creatine phosphokinase	1(33%)	2(50%)	0	3(11%)	Grades 1, 2
Fatigue	1(33%)	0	0	3(11%)	Grades 1, 2
Anxiety	0	0	0	2(13%)	Grade 1

Treatment administered every 12 h

^a^Preferred terms mapped to Medical Dictionary for Regulatory Activities version 16.0

^b^Grades assessed according to Common Terminology Criteria for Adverse Events version 4.0

^c^Includes the preferred terms rash maculopapular and dermatitis acneiform

Two treatment-related TEAEs, upper gastrointestinal hemorrhage and increased hepatic enzyme, were grade 3 and both were considered DLTs at the 40 mg Q12H dose level. No grade 4 events were considered possibly related to study treatment; thus, none were considered DLTs. Because of the DLTs observed at the 40 mg Q12H dosage, the MTD for use in part B was defined as 30 mg Q12H. Serious adverse events (SAEs) were reported in 9 patients (33.3%): 4 patients (33.3%) in part A and 5 patients (33.3%) in part B. In part A, the reported SAEs included large intestinal obstruction, lower gastrointestinal hemorrhage, small intestinal obstruction, pneumonia, and hypercalcemia (all in the same patient); peptic ulcer, hypovolemia, and hypotension (all in the same patient); sepsis; and upper gastrointestinal hemorrhage. In part B, the reported SAEs included staphylococcal pneumonia and dyspnea (both in the same patient), pericardial infusion, abdominal pain, rectal hemorrhage, and convulsion. The only SAE considered possibly related to study drug was the upper gastrointestinal hemorrhage reported in part A in a patient treated with 40 mg Q12H.

Six patients died during the study. One patient (part A, 30 mg Q12H) died during the treatment period of progressive disease, and five patients died of progressive disease during the 30-day follow-up period. It was the investigator’s opinion that none of these deaths were caused by TEAE.

### Pharmacokinetics

**Fig. 1:**
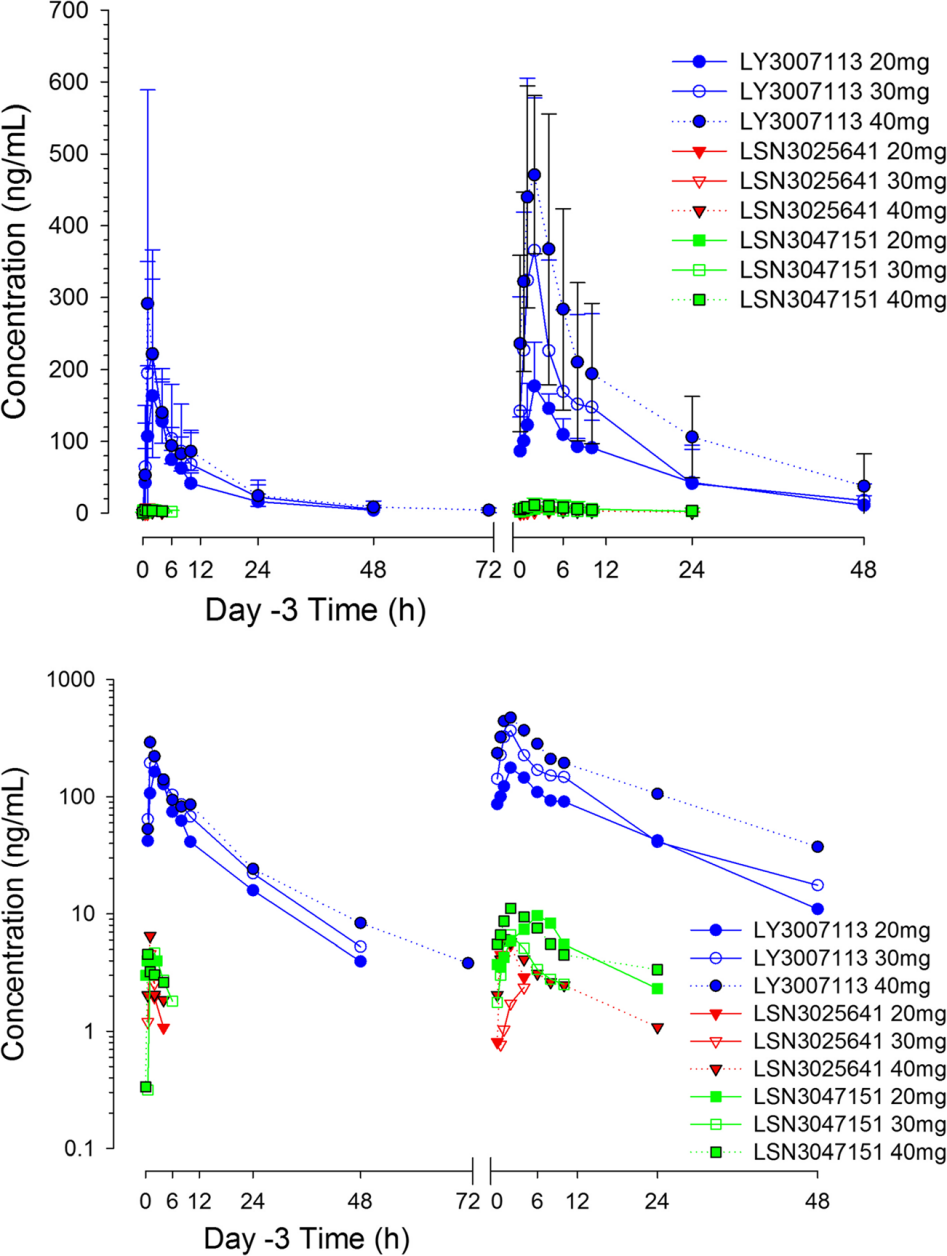
Arithmetic mean and SD plots per dose levels for LY3007113, LSN3025641, and LSN3047151 after single dose (Day −3) and repeated dose (Day 28) administration of LY3007113—linear scale (top panel) and semilog scale (bottom panel)

Mean concentration-versus-time profiles for LY3007113 and its metabolites LSN3025641 and LSN3047151 are presented in Fig. 1. Selected PK parameters for these 3 entities after either single or repeated administration of LY3007113 are summarized in Table 3. The mean t_max_ for LY3007113 was approximately 2 h (range, 0.5–6 h) after both single and repeated dosing. On cycle 1 day 28, the estimated geometric mean t_1/2_ was approximately 10 h (geometric coefficient of variation [geo CV], 46%; range, 5–27 h). Therefore, as expected with this value of t_1/2_, the LY3007113 accumulation ratio was approximately 1.8. The geometric means for apparent clearance and apparent volume of distribution were 14 L/h (geo CV, 56%) and 179 L (geo CV, 50%). The t_1/2_, apparent clearance, volume of distribution, and the metabolite-to-parent exposure ratios were similar after a single dose (day −3) and after one cycle of Q12H dosing (day 28).

**Table 3:** Noncompartmental pharmacokinetic parameter summary following oral administration of LY3007113 in either single dose (Day −3) or repeated doses (Day 28)—all PK population

	Geometric Mean (%CV)					
	Day −3			Day 28		
	20 mg (*n* = 3)	30 mg (*n* = 18)	40 mg (*n* = 4)	20 mg Q12H (*n* = 3)	30 mg Q12H (*n* = 18)	40 mg Q12H (*n* = 4)
C_max_, ng/mL	197 (3)	247 (49)	296 (66)	178 (31)	386 (64)	489 (31)
t_max_,^a^ hr	2.05 (1.00–4.00)	2.00 (0.52–6.00)	1.54 (1.02–4.00)	2.02 (2.00–4.08)	1.51 (1.00–4.00)	2.10 (1.00–4.03)
AUC_τ_, ng**·**hr./mL	987 (7)	1210 (48)	1380 (26)	1360 (23)		2070 (68)
AUC_(0–24)_,_ss_, ng**·**hr./mL	NC	NC	NC 2710	(23)	4140(68)	6530 (46)
AUC_(0-inf)_,^j^ ng**·**hr./mL	1560 (25)	1810 (67)	2480(32)	2580(64)	3220(92)	6590 (65)
CL/F, L/h	12.8 (25)	16.5 (67)	16.2(32)	14.8(23)	14.5(68)	12.3 (46)
V_ss_/F, L	168 (15)	181(39)	232(31)	232(36)	161(48)	204 (69)
t_1/2_,^b^ hr	10.8 (7.77–15.9)	8.85(3.65–15.7)	11.4 (7.9–16.8)	10.8 (8.01–15.9)	9.21(4.78–14.7)	14.0 (8.43–27.1)
R_A_,^c^ ratio	NC	NC	NC	1.54 (27)	1.66 (49)	2.37 (54)
LI,^d^ ratio	NC	NC	NC	0.871 (8)	1.08 (38)	1.32 (38)
M1:P,^e^ ratio	0.0110^i^	0.00817^k^ (163)	0.00972^g^ (28)	0.0129^h^ (122)	0.00972^m^ (82)	0.00969 (49)
M2:P,^f^ ratio	0.0290^h^ (80)	0.0154^l^ (117)	0.00941 (131)	0.0300 (238)	0.0193^m^ (48)	0.0193 (73)

*Abbreviations*: *AUC*_*(0–24),ss*_, area under the concentration-versus-time curve from time 0 to 24 h at steady state (as doubling AUC_(0–12),ss_); *CL/F*, apparent total body clearance after extravascular administration; *C*_*max*_, maximum plasma concentration; *%CV*, coefficient of variation; *LI*, linearity index; *M1*, LSN3025641; *M2*, LSN3047151; *NC*, not calculable; *P*, LY3007113; *RA*, accumulation ratio; *τ*, dosing interval 12 h; *ss*, steady state; *t*_*1/2*_, terminal half-life; *t*_*max*_, time to reach C_max_; *V*_*ss*_ */F*, apparent volume of distribution at steady state after extravascular administration

^a^Median (range)

^b^Geometric mean (range)

^c^Accumulation ratio AUC_(0–24)_ (Day 28) /AUC_(0–24)_ (Day −3)

^d^Linearity index AUCτ (Day 28) / AUC_(0-inf)_ (Day −3), where τ = 12 h

^e^LSN3025641 AUC_(0-inf)_ / LY3007113 AUC_(0-inf)_ (Day −3) or LSN3025641 AUCτ / LY3007113 AUCτ (Day 28)

^f^LSN3047151 AUC_(0-inf)_ / LY3007113 AUC_(0-inf)_ (Day −3) or LSN3047151 AUCτ / LY3007113 AUC τ (Day 28)

^g^ *n* = 3

^h^ *n* = 2

^i^ *n* = 1

^j^Based on only 1 dose received on Day 28

^k^ *n* = 14

^l^ *n* = 15

^m^ *n* = 11

In dose-proportionality analyses for the 20-mg to 40-mg dose range in this study, LY3007113 exhibited dose-normalized means ratios of 0.75 (90% CI: 0.41, 1.35; *n* = 25) after a single dose and 1.38 (90% CI: 0.70, 2.71; *n* = 19) after repeated dosing (Table 4). The same trend was observed for AUC_(0–12)_ and AUC_(0-inf)_. Because the 90% CI for the ratio of dose-normalized means for all dose-proportionality assessments always included 1, these findings do not suggest a large departure from linearity with an approximately dose-proportional increase in exposure across this range of doses.

**Table 4:** Dose proportionality assessment of Plasma LY3007113 over the 20- to 40-mg dosing range studied—all PK population

PK parameter^a^	Ratio of dose-normalized geometric means (90% CI)	Increase in exposure per dose doubling	%CV
Day −3 (single dose)			
C_max_, ng/mL	0.75 (0.41, 1.35)	1.50 (0.83, 2.71)	43.0
AUC_(0–12)_, ng**·**hr./mL	0.70 (0.42, 1.18)	1.40 (0.83, 2.37)	41.6
AUC_(0-inf)_, ng**·**hr./mL	0.79 (0.39, 1.61)	1.58 (0.78, 3.22)	50.5
Day 28 (repeated dose)			
C_max_, ng/mL	1.38 (0.70, 2.71)	2.76 (1.40, 5.42)	47.7
AUC_(0–12),ss_, ng**·**hr./mL	1.20 (0.59, 2.44)	2.40 (1.18, 4.89)	53.4
AUC_(0-inf)_,_ss_, ng**·**hr./mL	1.27 (0.48, 3.33)	2.53 (0.96, 6.65)	64.3

*Abbreviations*: *AUC(*_*0–12*_ *)*, area under the concentration-versus-time curve from time 0 to 12 h; *AUC(*_*0-inf*_ *)*, area under the concentration-versus-time curve from time 0 to infinity; *ss*, steady state; *CI*, confidence interval; *C*_*max*_, maximum plasma concentration; *%CV*, coefficient of variation

^a^AUC and C_max_ parameters were transformed to the natural log scale for analysis, and results were transformed back into the original scale

### Pharmacodynamics

**Fig. 2:**
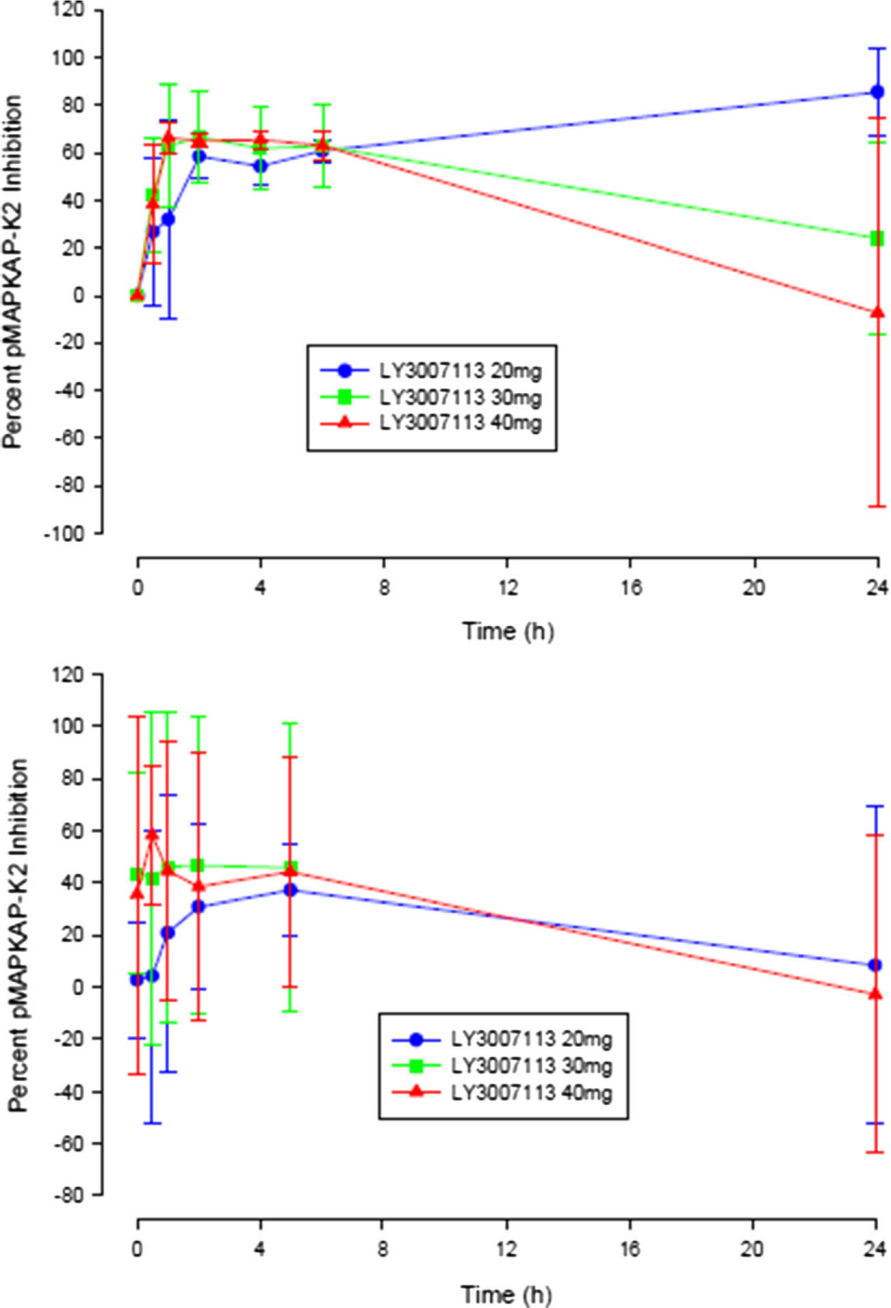
Mean (SD) phosphorylated MAPK-activated protein kinase 2 corrected stimulated molecules of equivalent fluorescein percentage inhibition profiles by LY3007113 dose level after single dose (top panel) and repeated doses (bottom panel)

For 22 of 24 patients in the 20-mg to 40-mg dosing range, maximum p-MAPKAP-K2 inhibition in PBMCs after a single dose exceeded 60% (Fig. 2). After repeated dosing, however, interpatient variability was much higher, and the mean p-MAPKAP-K2 inhibition was lower than after a single dose. The defined BED was not reached because neither maximal inhibition (80%) nor sustained minimal inhibition (60%) for up to 6 h after dosing was observed.

### Overall response rate

No tumor responses were seen in this study. In part B, 3 patients (23.1%) had a best response of stable disease for tumor types, including renal cell carcinoma, gastric adenocarcinoma, and epithelial ovarian carcinoma. In these patients, radiographically assessed stable disease was maintained for periods ranging from 58 days to 115 days. Ten patients (76.9%) had a best response of progressive disease for tumors including carcinoma (small cell lung, renal cell, breast, and adrenocortical), adenocarcinoma (rectum and prostate), squamous cell carcinoma (head and neck), cholangiocarcinoma, and sarcoma. Two patients discontinued early and were not assessed for response.

## Discussion

This phase 1 study evaluated safety, PK, PD, and tumor response in patients with advanced cancer after oral administration of LY3007113. Nine patients (33.3%) experienced an SAE, and 18 patients (66.7%) experienced at least one TEAE. The MTD was 30 mg Q12H, although the expected dose ranged from 20 mg to 200 mg. Because of toxicities observed early in the study, such as tremors (33.3%) and rash (22.2%), dose escalation was stopped at 40 mg Q12H; thus, the predetermined targets of either maximal inhibition (80%) or sustained minimal inhibition (60%) for up to 6 h after dosing were not achieved. The relationship between exposure and safety is not clear because the DLTs observed at 40 mg Q12H (upper gastrointestinal haemorrhage and increased hepatic enzyme) occurred in patients whose relative exposures were no higher than those of other patients receiving the same dose (Table 2).

The estimated t_1/2_ of LY3007113 in humans provides flexibility for dosing of LY3007113 either Q12H or every 24 h. Administration Q12H was selected primarily to minimize the difference between peak and trough levels at steady state, thus maintaining a more consistent level of target inhibition. The PK findings in this study showed LY3007113 PK and metabolism to be time independent. They also suggest that both active metabolites, LSN3025641 and LSN3047151, contributed negligibly to LY3007113 activity: the observed exposure to these metabolites was less than 3% of the exposure to the parent LY3007113. The in vitro potency of the metabolites was either one-half or one-third of the in vitro potency of the parent LY3007113.

The PD findings regarding p-MAPKAP-K2 inhibition in PBMCs suggest that the predicted BED of LY3007113, which was based on preclinical data, was not reached: we observed neither maximal inhibition (80%) nor sustained minimal inhibition (60%) for up to 6 h after dosing. In addition, no explanation was found for the pattern of higher interpatient variability and lower inhibition seen after repeated versus single dosing. Given the high incidence of tremors, fatigue, and rash, it was not clinically feasible to continue with dose escalation as planned. Thus, these factors may have contributed to a lack of clinical responses.

## Conclusion

The recommended dosage of LY3007113 was established at 30 mg Q12H, although a biologically effective dose was not achieved due to tolerability concerns. With only 3 of 27 patients (11.1%) continuing treatment after the first radiographic assessment, further clinical study of this compound is not planned.

## References

[1] Reinhardt HC, Aslanian AS, Lees JA, Yaffe MB (2007). p53-deficient cells rely on ATM- and ATR-mediated checkpoint signaling through the p38MAPK/MK2 pathway for survival after DNA damage. Cancer Cell.

[2] del Barco B, Nebreda AR (2012). Roles of p38 MAPKs in invasion and metastasis. Biochem Soc Trans.

[3] Kotlyarov A, Neininger A, Schubert C, Eckert R, Birchmeier C, Volk HD, Gaestel M (1999). MAPKAP kinase 2 is essential for LPS-induced TNF-alpha biosynthesis. Nat Cell Biol.

[4] Lewis AM, Varghese S, Xu H, Alexander HR (2006). Interleukin-1 and cancer progression: the emerging role of interleukin-1 receptor antagonist as a novel therapeutic agent in cancer treatment. J Transl Med.

[5] Balkwill F, Mantovani A (2005). Inflammation and cancer: back to Virchow?. Lancet.

[6] Arechederra M, Priego N, Vázquez-Carballo A, Sequera C, Gutiérrez-Uzquiza Á, Cerezo-Guisado MI, Ortiz-Rivero S, Roncero C, Cuenda A, Guerrero C, Porras A (2015). p38 MAPK down-regulates fibulin 3 expression through methylation of gene regulatory sequences: role in migration and invasion. J Biol Chem.

[7] Tate CM, Blosser W, Wyss L, Evans G, Xue Q, Pan Y, Stancato L (2013). LY2228820 dimesylate, a selective inhibitor of p38 mitogen-activated protein kinase, reduces angiogenic endothelial cord formation in vitro and in vivo. J Biol Chem.

[8] Alspach E, Flanagan KC, Luo X, Ruhland MK, Huang H, Pazolli E, Donlin MJ, Marsh T, Piwnica-Worms D, Monahan J, Novack DV, McAllister SS, Stewart SA (2014). p38MAPK plays a crucial role in stromal-mediated tumorigenesis. Cancer Discov.

[9] Coulthard LR, White DE, Jones DL, McDermott MF, Burchill SA (2009). p38(MAPK): stress responses from molecular mechanisms to therapeutics. Trends Mol Med.

[10] Hideshima T, Akiyama M, Hayashi T, Richardson P, Schlossman R, Chauhan D, Anderson KC (2003). Targeting p38 MAPK inhibits multiple myeloma cell growth in the bone marrow milieu. Blood.

